# Mortality and excess risk in US adults with pre-diabetes and diabetes: a comparison of two nationally representative cohorts, 1988–2006

**DOI:** 10.1186/1478-7954-11-3

**Published:** 2013-02-28

**Authors:** Andrew Stokes, Neil K Mehta

**Affiliations:** 1 Population Studies Center, University of Pennsylvania, 3718 Locust Walk, 239 McNeil Building, Philadelphia, PA, 19104-6298, USA; 2 Department of Global Health, Emory University, 1518 Clifton Road, Atlanta, GA, 30322, USA

**Keywords:** Pre-diabetes, Diabetes, Dysglycemia, Diabetes management, Mortality, Mortality change

## Abstract

**Background:**

There is strong evidence on the efficacy of behavioral modification and treatment for reducing diabetes incidence and diabetes-related morbidity and mortality in persons with pre-diabetes and diabetes. But the extent to which the evidence has translated into gains in health in these population sub-groups in the US is unclear. Monitoring national diabetes-related mortality levels over time is important for evaluating the effectiveness of the US health system response to diabetes.

**Methods:**

We identified individuals with pre-diabetes and diabetes using Hemoglobin A1c. Two consecutive periods for investigating differences in mortality according to categories of glycemia were derived using nationally representative survey data on US adults ages 35–74 from subsequent rounds of the National Health and Nutrition Examination Survey (1988–1994 and 1999–2002). Age-standardized mortality rates were calculated for individuals with pre-diabetes and diabetes and proportional hazards models were used to assess change in the relative risks of dysglycemia (pre-diabetes and diabetes) adjusting for multiple confounding factors.

**Results:**

Age-standardized mortality rates in individuals with pre-diabetes and diabetes showed no statistically significant change between 1988–2001 and 1999–2006. In individuals with pre-diabetes, mortality rates were 11.19 and 14.02 deaths per 1,000 person-years in the early and later period, respectively. The corresponding values for individuals with diabetes were 20.34 and 20.82 deaths per 1,000 person-years. In contrast, the absolute level of mortality in the normo-glycemic population declined significantly between 1988–2001 and 1999–2006 (7.81 to 6.04; p for difference < 0.05). Adjusting for social and demographic variables, smoking and body mass index in a multivariate analysis, the hazard ratio of dysglycemia increased from 1.62 (95% CI: 1.36–1.93) in 1988–2001 to 2.36 (95% CI: 1.70–3.27) in 1999–2006 (p for difference < 0.05).

**Conclusions:**

We find no evidence of declines in excess mortality in persons with dysglycemia between 1988–2001 and 1999–2006, a result that was robust to adjustment for social and demographic variables, smoking and body mass index. In the context of long-term secular declines in mortality in the US population, our findings suggest that individuals with pre-diabetes and diabetes should be an important focus of future interventions aimed at improving population health in the US.

## Background

Diabetes mellitus is a leading cause of death in the US [1]. Clinical trial data have demonstrated the efficacy of lifestyle and pharmacological interventions for reducing the incidence and complications of diabetes [2-5]. Concurrent management of cardiovascular risk factors in individuals with diabetes has shown to result in particularly large improvements in health outcomes [6,7].

Despite the demonstrated efficacy of primary and secondary diabetes prevention strategies, achieving improved population health outcomes in practice requires aggressive screening and overcoming the complexities of managing diabetes and associated conditions. Diabetes management requires long-term medical care, input and coordination across health providers, patient self-education and motivation and addressing multiple behavioral, metabolic and cardiovascular risk factors simultaneously [8]. Previous studies have used diabetes as an indicator of health systems performance in recognition that successful implementation of these strategies relies centrally on high quality and coordinated health care [9].

Evidence on the processes of care for individuals with diagnosed diabetes for the 1990s and early 2000s indicates that treatment rates have improved, but that cardiovascular risk factor control remains sub-standard [10-12]. With respect to glycemic control, little to no change was observed over this period [11,12]. Information on mortality trends in individuals with pre-diabetes and diabetes is critical for a full assessment of progress towards the more effective control of diabetes-related outcomes in the US population. Trends have been assessed in three previous nationally representative studies of the US population [13-15]. In all of the previous studies, individuals with diabetes were identified using self-reported data on having ever received a diagnosis of diabetes by a health care provider. However, diagnostic criteria [16] and levels of screening [17,18] have changed over time, affecting selection into the population with diagnosed diabetes and complicating interpretation of trends. Also, because diabetes is undiagnosed in a third to a fifth of US adults with the condition depending on the criterion used [19], trends identified in prior studies may not reflect trends occurring among the overall diabetic population. Information on trends in the overall population with diabetes is important for a more complete assessment of the health system response to diabetes, the success of which depends both on diagnosis and control of diabetes.

In this study, we investigate change over time in absolute and excess mortality rates in individuals with pre-diabetes and diabetes (collectively, ‘dysglycemia’) during the period 1988–2006, which is the most recent period for which nationally representative data are available. We extend the focus to individuals with pre-diabetes as this group is at high risk for developing diabetes and its complications and is considered a high-priority group for intervention [8]. We use a clinical diagnostic measure, Hemoglobin A1c (HbA1c), to identify individuals with dysglycemia and adjust for key confounding factors in a multivariate analysis of mortality change.

## Methods

We established two consecutive periods for investigating mortality change according to diabetes status using data from the National Health and Nutrition Examination Survey (NHANES). NHANES is a series of nationally representative surveys of the non-institutionalized U.S. population conducted by the National Center for Health Statistics (NCHS). The survey includes an examination component in which extensive medical data are collected by trained nurses in mobile clinics or in home-visits and is linked to the National Death Index, a computerized index of death record information maintained by the NCHS [20]. The earlier period in our analysis was constructed using data from 1988–1994 (NHANES III) and the later period using data from 1999–2002 (NHANES CTS). To reduce bias that may result from differential follow-up across the two mortality periods, we restricted mortality follow-up in NHANES III to 7.7 years, the maximum follow-up available in NHANES CTS. The resulting periods for our analysis of mortality change were 1988–2001 for NHANES III and 1999–2006 for NHANES CTS.

We restricted the sample to individuals ages 35–74 who underwent examination and had non-missing information on diabetes and death (3.7% and 0.1% of persons were missing information on each of these items). The resulting sample included 9,210 observations on the earlier cohort and 5,470 observations on the later cohort. We defined diabetes on the basis of the HbA1c test, which is standardized to the methods of the Diabetes Control and Complications Trial in both periods [21]. A distinct advantage of HbA1c is that it reflects average glycaemia over a prolonged period of time and exhibits greater pre-analytic stability and lower intra-individual variation than other diagnostic markers, such as fasting plasma glucose (FPG) [22]. We used the most recent national American Diabetes Association (ADA) guidelines [8] to classify individuals into categories of non-diabetes (HbA1c less than 5.7%), pre-diabetes (HbA1c between 5.7% and 6.5%) or diabetes (HbA1c more than 6.5%). We further classified as diabetic persons whose HbA1c values were below 6.5% but who reported use of an oral hypoglycemic agent or insulin. When referring collectively to individuals with pre-diabetes and diabetes, we use the term ‘dysglycemia’. To minimize bias, we did not consider information on physician-diagnosed diabetes because diagnostic criteria changed over the period of analysis [23]. Approximately 1% of normo-glycemic individuals not currently taking medications for diabetes reported a previous diagnosis of the condition.

We calculated age-standardized mortality rates separately by diabetes status and period as the ratio of deaths to person-years of follow-up, in each case using the year 2000 Census population as the standard. Within each period, we calculated relative risks comparing pre-diabetes and diabetes categories to no diabetes.

We also implemented Cox proportional hazards models to estimate mortality risks associated with pre-diabetes and diabetes adjusted for covariates and to test for the significance of differences in these risks across periods. Attained age was used as analysis time. We imputed missing covariate values using multiple imputation with Amelia II software [24,25]. The imputation model included all model covariates, including transformations. Overall, 1.1% of persons were missing information on BMI, 0.5% on education and 0.1% on smoking. Changes in the hazard ratios were estimated by introducing an interaction into the model between diabetes status and an indicator variable for period. We initially assessed whether the proportionate change in the hazard ratios differed across the two dysglycemic groups. The two values were statistically indistinguishable from one another; therefore in subsequent analysis we pooled individuals with pre-diabetes and diabetes into a dysglycemic group. We additionally examined sex-specific differences in the interaction, but found no statistical differences by sex so we present results for the sexes combined. In Model 1, we adjusted for social and demographic covariates, including sex, race/ethnicity (indicators for non-Hispanic black and Hispanic ethnicity) and education (less than high-school education, high-school education and more than high-school education). In Model 2, we additionally adjusted for smoking using categories of current, former and never smoker. In Model 3, we extended Model 2 by including linear and quadratic terms for body mass index (BMI) [26]. We adjusted for smoking and BMI as they are strong correlates of diabetes and previous research suggests that smoking cessation may increase the risks of developing diabetes [27,28].

We introduced interaction terms between each covariate and analysis time into the model to check the validity of the proportional hazards assumption. As the assumption was not met for some covariates (black/non-black and education), we re-estimated Models 1–3 retaining these particular interactions. We found that results were not sensitive to their inclusion. All results incorporated sampling weights which capture unequal probabilities of selection and non-response adjustments and accounted for the complex survey design of NHANES. Analyses were conducted using STATA 12 (StataCorp, Texas, USA).

## Results

Table 1 shows descriptive characteristics of the US adult population by diabetes status and period. In both periods, individuals in the dysglycemic groups were generally older, less educated, and more likely to be Hispanic or non-Hispanic black compared to those without diabetes. Changes over time in the distribution of smoking status and body mass index (BMI) differed across the three groups. The proportion of current smokers declined significantly over time for the pre-diabetic group (37.63% to 22.13%; p < 0.05), but not for the non-diabetic (25.43% to 23.91%) and diabetic groups (22.01% to 19.13%). Larger increases in the percentage of never smokers were observed in persons with dysglycemia compared to those without. The proportion with severe obesity (BMI ≥ 35 kg/m2) increased in all groups, but somewhat larger absolute increases were observed in individuals with dysglycemia (differential increase of 5% [95% CI 1-10%; p < 0.05]). The proportion with severe obesity was three times higher among individuals with diabetes than among those without diabetes in the more recent period. Table 1 also shows mean HbA1c levels, which remained essentially unchanged between periods for individuals with pre-diabetes and diabetes.

**Table 1:** Descriptive statistics of the U.S. adult population ages 35-74 by diabetes status and period

	**No Diabetes**		**Pre-Diabetes**		**Diabetes**	
	**NHANES III**	**NHANES CTS**	**NHANES III**	**NHANES CTS**	**NHANES III**	**NHANES CTS**
Sample size	6,277	4,014	1,679	660	1,254	796
Mean age, y	49.45	49.47	56.19	55.52	57.11	56.99
Hispanic,%	7.62	10.84	10.26	15.65	11.25	16.91
Non-Hispanic Black,%	7.32	8.71	20.07	16.67	17.69	17.79
Education,%						
Less than High School	20.99	17.66 †	35.63	29.06	44.35	37.61
High School	33.21	24.45 †	35.56	28.52 †	33.15	24.84 †
More than High School	45.80	57.89 †	28.82	42.42 †	22.51	37.55 †
Smoking Status,%						
Current	25.43	23.91	37.63	22.13 †	22.01	19.13
Former	31.51	28.51 †	30.04	33.85	40.98	34.56
Never	43.06	47.58 †	32.33	44.02 †	37.02	46.31 †
Mean BMI^a^ (kg/m^2^)	26.69	27.73 †	28.85	31.74 †	31.41	32.86 †
BMI^a^ category (kg/m^2^),%						
30 to 35	15.09	17.41 †	20.34	31.98 †	28.25	25.68
More than 35	6.88	10.48 †	15.33	24.27 †	26.20	33.15 †
Mean HbA1c	5.16	5.24 †	5.99	5.97	8.11	8.00

BMI: body mass index; HbA1c: Hemoglobin A1c; a. BMI derived from measure data on height and weight; No Diabetes is defined as HbA1c less than 5.7%; Pre-Diabetes is defined as HbA1c between 5.7 and 6.5%; Diabetes is defined as HbA1c above 6.5% or on treatment for diabetes. Sources: National Health and Nutrition Examination Survey (NHANES) III (1988–1994) and continuos (CTS) (1999–2002).

† Significantly different (p < 0.05) compared with NHANES III.

Table 2 shows age-standardized mortality rates and relative risks by diabetes status and period. Whereas the age-standardized mortality rate in the normo-glycemic population declined significantly between 1988–2001 and 1999–2006 (7.81 to 6.04; p for difference < 0.05), absolute levels of mortality among individuals with pre-diabetes and diabetes showed no statistically significant change. The mortality rate increased from 11.19 to 14.02 deaths per 1,000 person-years in individuals with pre-diabetes and from 20.34 to 20.82 deaths per 1,000 person-years in individuals with diabetes. Relative risks of mortality grew across periods for both individuals with pre-diabetes (1.43 to 2.32) and those with diabetes (2.60 to 3.45). These changes were equivalent to increases of 62% and 32% in the relative risks and were not statistically significant.

**Table 2:** All-cause mortality rates (per 1,000 person-years) and relative risks for the US adult population by diabetes status and period

	**NHANES III**	**NHANES CTS**
	**(1988-2001)**	**(1999-2006)**
***No Diabetes***		
Mortality Rate	7.81 (6.70-8.93)	6.04 (4.77-7.32) †
Relative Risk	1.00	1.00
***Pre-Diabetes***		
Mortality Rate	11.19 (8.38-14.00)	14.02 (8.47-19.58)
Relative Risk	1.43 (1.12-1.74)	2.32 (1.24-3.40)
***Diabetes***		
Mortality Rate	20.34 (16.00-24.67)	20.82 (13.10-28.54)
Relative Risk	2.60 (1.91-3.29)	3.45 (2.02-4.87)
		
Deaths	960	341
Person-years	67,526	30,989

HbA1c: Hemoglobin A1c; No Diabetes is defined as HbA1c less than 5.7%; Pre-Diabetes is defined as HbA1c between 5.7 and 6.5%; Diabetes is defined as HbA1c above 6.5% or on treatment for diabetes. Sample includes persons ages 35-74 at baseline. Entry years are 1988-1994 with follow-up through 2001 for the earlier period and 1999-2002 with follow-up through 2006 for the later period. Mortality rates are age-standardized to the year 2000 Census population using age groups 35-54, 55-69 and 70-84. Relative risks are based on age-standardized mortality rates and are otherwise unadjusted. All estimates are weighted and account for complex survey design. Sources: National Health and Nutrition Examination Survey (NHANES) III and continuous (CTS).

^†^Significantly different (p < 0.05) compared with NHANES III.

The differences in mortality across the two periods in Table 2 may partially reflect changes in background factors related to both diabetes and mortality, such as race/ethnicity, education, smoking and BMI. Table 3 shows results from multivariate proportional hazards models adjusting for these covariates. In these models, the hazard ratio of the ‘dysglycemia x period’ term expresses the proportionate change in the hazard ratio associated with dysglycemia from the earlier to the later mortality period (1988–2001 to 1999–2006). In Model 1, adjusting for social and demographic factors, the hazard ratio of dysglycemia was 1.63 (95% CI: 1.38-1.92) in 1988–2001. The hazard ratio of the two-way interaction indicated that the relative risks increased by 34% across the two periods; however the difference was not significant. Multiplying these two terms yields a hazard ratio for dysglycemia of 2.18 (95% CI: 1.57-3.04) in the later period (1999–2006).

**Table 3:** Hazard ratios predicting mortality from all causes

	**Model 1**		**Model 2**		**Model 3**	
	**Hazard Ratio**	**95% CI**	**Hazard Ratio**	**95% CI**	**Hazard Ratio**	**95% CI**
Sex						
Women	1.00		1.00		1.00	
Men	1.54 ^***^	(1.34-1.76)	1.30 ^***^	(1.13-1.50)	1.33 ^***^	(1.16-1.53)
Race/ethnicity						
Other	1.00		1.00		1.00	
Hispanic	0.76	(0.57-1.02)	0.83	(0.62-1.12)	0.84	(0.63-1.13)
Non-Hispanic Black	1.48 ^***^	(1.21-1.82)	1.51 ^***^	(1.23-1.84)	1.49 ^***^	(1.22-1.81)
Education Level						
Less than High School	1.00		1.00		1.00	
High School	0.78 ^**^	(0.65-0.93)	0.79 ^**^	(0.66-0.94)	0.79 ^**^	(0.67-0.94)
More than High School	0.61 ^***^	(0.50-0.74)	0.68 ^***^	(0.56-0.83)	0.67 ^***^	(0.55-0.82)
Smoking						
Never Smoker			1.00		1.00	
Former Smoker			1.83 ^***^	(1.46-2.29)	1.83 ^***^	(1.46-2.39)
Current Smoker			2.90 ^***^	(2.24-3.75)	2.73 ^***^	(2.11-3.52)
BMI					0.87 ^***^	(0.82-0.93)
BMI Squared					1.00 ^***^	(1.00-1.00)
Period^a^	0.86	(0.65-1.13)	0.86	(0.66-1.13)	0.88	(0.67-1.15)
Dysglycemia^b^	1.63 ^***^	(1.38-1.92)	1.55 ^***^	(1.31-1.83)	1.62 ^***^	(1.36-1.93)
Dysglycemia X Period	1.34	(0.94-1.92)	1.45^*^	(1.02-2.07)	1.46 ^*^	(1.03-2.08)

a. Period is an indicator variable which takes a value of 1 in the later of the two periods in the analysis. Entry years are 1988-1994 with follow-up through 2001 for the earlier period and 1999-2002 with follow-up through 2006 for the later period; b. Dysglycemia sample includes persons with HbA1c ≥ 5.7% or on treatment for diabetes. References categories for categorical variables indicated by HR = 1.00; Sample includes persons ages 35-74 at baseline. All estimates are weighted and account for complex survey design.

Sources: National Health and Nutrition Examination Survey III and continuous.

^***^p < 0.001; ^**^ < 0.01; ^*^p < 0.05

When smoking was included in the model, we observed a statistically significant increase in the hazard ratio for dysglycemia across the two periods (HR = 1.45 for the interaction term; 95% CI: 1.02-2.07). In Model 2, the hazard ratio of dysglycemia was 1.55 (95% CI: 1.31-1.83) in 1988–2001 and 2.26 (95% CI: 1.64-3.11) in 1999–2006. The increase in the magnitude of the interaction term between Model 1 and Model 2 was due to both a slight decline in the hazard ratio in the early period (from 1.63 to 1.55) and a slight increase in the hazard ratio applicable to the later period (2.18 to 2.26). These changes are likely attributable to the changing association between smoking and diabetes status across time as shown in Table 1. Additional adjustment for BMI and BMI-squared had little effect on the magnitude of the estimated change (Model 3).

## Discussion

In this paper, we investigated mortality of individuals with dysglycemia relative to individuals with normo-glycemia and the extent to which the level of excess mortality associated with dysglycemia, adjusting for confounding factors, has changed over the period 1988–2006. Whereas the age-standardized mortality rate declined significantly between periods in the normo-glycemic population, mortality rates in individuals with pre-diabetes and diabetes showed no clear evidence of change. In our multivariate analysis, dysglycemia was associated with a non-significant increase in mortality over time in a model that adjusted for socio-demographic characteristics. However, a more pronounced and statistically significant increase in excess risk was observed upon further adjustment for smoking, suggesting that secular trends in smoking and its distribution by diabetes status may partially obscure change in the mortality risks of dysglycemia. Our multivariate results, based on adjustment for social and demographic variables, smoking and body mass index, indicated a 46% increase in the relative risk of dysglycemia for the US adult dysglycemic population.

Our findings follow on two previous nationally representative studies that have documented increasing gaps in mortality between individuals with and without diabetes. Excess mortality risks for those who reported a diabetes diagnosis were found to have increased over the 1970s and 1980s [13]. In a subsequent study that included more recent cohort experience from years 1988–1994 and mortality follow-up through 2001 excess mortality appeared to increase among women but not in men with diabetes relative to 1976–1980 [14].

Diabetes-related mortality trends have also been investigated using data from the Framingham Heart Study [29]. This study used clinically measured glucose levels and found that excess mortality declined between 1950–1979 and 1976–2005. However, in a sensitivity analysis in which the authors investigated trends in a more recent and narrow time interval (1971 to 2000), no statistically significant declines in absolute or excess all-cause mortality were identified.

Our findings contrast with those of a recent study that investigated three-year US mortality rates across four cohorts (1997–1998, 1999–2000, 2001–2002 and 2003–2004) using data from the National Health Interview Survey [15]. This study found declines in excess mortality in individuals with previously diagnosed diabetes between 1997–1998 and 2003–2004. However, as one-third to one-fifth of the diabetic population is undiagnosed [19], trends identified in the prior study may not be reflective of those for the entire adult population with diabetes. Criteria for diagnosis and screening of diabetes changed over the period of the analysis, further complicating interpretation of trends based on self-reported data for defining diabetes. With respect to diagnostic criteria, in 1997 the ADA established new guidelines for the diagnosis of diabetes, based on FPG, which lowered the threshold of diagnosis from 140 mg/dL to 126 mg/dL [23]. Any changes in the severity distribution of diabetes induced by the change in guidelines, which are likely to have been implemented gradually over time, would have likely biased trends estimates using self-reported data. Secular increases in screening may have also affected the composition of the population with diabetes over the two periods, by increasing the proportion of diabetes cases detected early. However, in a sensitivity analysis, the findings of the prior study were found to be robust to the exclusion of individuals with recent onset diabetes (2 years or less) [15].

Our consideration of individuals with pre-diabetes as well as those with diabetes may have also contributed to the differences in findings between the two studies. Given the absence of population-based screening in the US, individuals with non-diabetic dysglycemia may have benefited less from health services than individuals with diabetes, leading to lower likelihoods of mortality improvements over time in that sub-population. Finally, differences between studies could relate to the fact that HbA1c may identify a somewhat different subset of individuals with diabetes than FPG and other diagnostic measures in common use [19].

Our results also differ with evidence from the UK, where inequalities in mortality between individuals with and without diabetes are reported to have declined in the interval 1996–2006 [30]. Health systems factors may partially account for these differences [31]. One study points to improved management of diabetes in the UK compared to the US [32].

A key strength of our analysis is our use of HbA1c criteria rather than self-report to identify individuals with dysglycemia. To our knowledge, this is the first study to use an objective blood marker in the examination of national diabetes-related mortality change in the US. An additional strength of our study is that we included adjustments for key confounding variables, such as obesity and smoking, in our multivariate analysis. Our analysis was limited by small sample sizes, which prevented an investigation of the role of specific causes of death or a more detailed exploration of the mechanisms underlying increased relative mortality risks between the two periods. For example, one factor that may have contributed is an increase in the incidence of diabetes among individuals with dysglycemia across the two time periods. Although we detected no significant differences in the extent of mortality change between persons with pre-diabetes vs. diabetes or in men vs. women, there may be sub-group heterogeneity that we could not discern given limited statistical power. Future research should explore mortality trends in specific population sub-groups defined by diabetes and sex, investigate trends across cohorts more narrowly defined in calendar years and explore the sensitivity of trends to the use of alternate diagnostic measures of diabetes, such as FPG. We cannot rule out the possibility that our findings are affected by residual confounding. For example, adjustment for BMI at baseline may not have fully captured patterns of change in the duration of obesity [33], central adiposity and other features of the metabolic syndrome.

In conclusion, we find no evidence that excess mortality in US adults with dysglycemia decreased over the interval 1988–2006. These findings were not explained by age, race or ethnicity, education, smoking or rising BMI levels in persons with dysglycemia. When contrasted with the long-term secular declines in all-cause and cardiovascular mortality in the US population [34], the lack of improvement in mortality in the dysglycemic population is concerning and suggests that individuals with pre-diabetes and diabetes should be an important focus of future interventions aimed at improving population health in the US.

## Competing interests

The authors declare that they have no competing interests.

## Authors’ contributions

AS and NKM conceived of the study’s idea. AS compiled the data, conducted the statistical analysis and wrote the first draft of the paper. NKM contributed to the statistical analysis and writing of the manuscript. Both authors read and approved the final manuscript.
